# The Needs of Incarcerated Pregnant Women: A Systematic Review of Literature

**DOI:** 10.30476/IJCBNM.2021.89508.1613

**Published:** 2022-01

**Authors:** Somayeh Alirezaei, Robab Latifnejad Roudsari

**Affiliations:** 1 Student Research Committee, Mashhad University of Medical Sciences, Mashhad, Iran; 2 Nursing and Midwifery Care Research Center, Mashhad University of Medical Sciences, Mashhad, Iran; 3 Department of Midwifery, School of Nursing and Midwifery, Mashhad University of Medical Sciences, Mashhad, Iran

**Keywords:** Need assessment, Pregnancy, Prison, Women

## Abstract

**Background:**

With increase in the number of female prisoners, it seems necessary to follow up the conditions of pregnant women in prison in order to identify their needs and provide healthcare and social services to improve their health accordingly. Therefore, a systematic review was conducted to examine the needs of incarcerated pregnant women.

**Methods:**

In this systematic review, we searched the databases including PubMed, Scopus, Web of Science, EMBASE, PsycINFO, and the Cochran Library. All studies including cross-sectional, retrospective, and prospective cohorts as well as case series, which addressed the needs and expectations of incarcerated pregnant women, were included in this review. Two reviewers independently evaluated the retrieved articles, the discrepancies were discussed, and a consensus was achieved.

**Results:**

31 eligible studies consisting of 5435 incarcerated pregnant women were included in the review. The needs of incarcerated pregnant women comprised six general categories: healthcare needs including prenatal, labor, delivery, and postpartum services; educational needs on pregnancy, childbirth, and parenting; the support needs to be provided by government agencies, social workers, and doula services; the need for psychological counseling services; nutritional needs during pregnancy; and the needs related to the substance abuse management.

**Conclusion:**

The needs of incarcerated pregnant women included healthcare, educational, supportive, counseling, and nutritional needs as well as those related to the substance abuse management. Identifying these needs can be useful in developing accurate and appropriate policies and programs to promote the health status of this vulnerable group.

## INTRODUCTION

With increase in the number of female prisoners all around the world, pregnancy in this population has also become an important concern. Many organizations do not provide statistical reports on pregnancies in prison, and most of them do not have a routine process for recording and collecting the data. ^1^ Published reports indicate that about three to four hundred women are pregnant at the beginning of their imprisonment in the US state and federal prisons. ^2 - 4^ Other reports show between six and ten percent of pregnancies are in the prison all around the world. ^5^ According to the last report in Iran, the number of female prisoners is on increase, accounting for 3.1 percent of the total prison population. The global growth rate of this population has increased from 7.2% in 2006 to 8.8% in 2014. ^1^

In the female prisoner population, pregnant women are identified as a high-risk and special group. ^6^ The consequences of pregnancy in prison indicate the need for these women to optimize and pay attention to their living conditions in prison. ^2^ Investigating the official UK government documents shows the special reproductive and psychosocial needs of pregnant prisoners. The personnel of prisons or hospital staff had subjected some of these women to some form of verbal, psychological, or physical violence during their stay in prison or during childbirth. Most of them, because of the lack of proper family or social relationships, needed support. Problems of being bound during labor and delivery processes added to their problems. Also after giving birth and returning to prison, they did not receive the required postpartum care, and more importantly, they were separated from their infant very soon. ^7^ World Health Organization in its “recommendations on antenatal care for a positive pregnancy experience” has highlighted that the needs of incarcerated pregnant women are not considered in the prisoner care program. ^8^ In Iran, also, despite the existence of health guidelines in the prison system, ^9^ no adequate attention is paid to the health of pregnant women. ^10^

These documents show that incarcerated pregnant women are vulnerable in the society, both inside the prison and after their release. In some ways, the needs of incarcerated pregnant women are not the same as those of other vulnerable women in a free society, because they have access to national and international clinical guidelines. ^11^ However, imprisonment impose unique restrictions on daily life plans, nutrition and diet, prenatal care, delivery, support, and contact with the newborn. ^12^

In a study on reproductive justice in the US prisons, it has been highlighted that for preventing further vulnerability of incarcerated pregnant women and meeting their health and fertility needs, prison policies and practices need to be improved and reformed In this study, it has also been emphasized that there are still many problems and deficiencies in relation to the pregnant women’s health in prison It is noteworthy that the organizational facilities are not enough to deal with the pregnancy issues in prisons. Also, the needs of pregnant women have not been properly understood. ^13^ Examination of the existing documents for the care of pregnant women in Iran showed that Examination of the existing documents for the care of pregnant women in Iran showed that the health promotion of incarcerated pregnant women was not prioritized in healthcare planning. In a review of international guidelines on healthcare for pregnant women in prison, it has been emphasized that there are currently gaps in the healthcare guidelines in many aspects; for upgrading these guidelines and providing comprehensive and complete care programs, it seems necessary to identify and classify the needs of incarcerated pregnant women through conducting a systematic review on this vulnerable population needs. ^14^ This information gap has caused disciplinary organizations to fail to achieve the optimum level of care and not have the required efficiency. ^15^ The health care provided to imprisoned pregnant women is of considerable public health importance, ^16^ and no systematic review has been found to address this salient issue. Because of the lack of data and the issues previously argued based on the relevant literature, the focus of the present review moved to an examination of the current worldwide situation in terms of healthcare needs for pregnant women in prison. Systematic reviews could identify, appraise, and summarize the results of all relevant studies over a health-related issue, so they would make the available evidence more accessible for program and policy development. ^17^ This review aimed to systematically summarize and critically evaluate the literature so that a clear understanding of the health needs is gained.

## METHODS

This study was performed based on the preferred reporting items for systematic reviews and meta-analysis (PRISMA) checklist for systematic reviews. ^18^ An intensive and regular search of English articles was conducted in databases of PubMed, Scopus, Web of Science, EMBAS, PsycINFO, the Cochran Library as well as the reference lists of the retrieved articles through hand searching between 1984 and 2020. The keywords included: (Pregnancy OR “Pregnant Women” OR “Prenatal Care” OR “Antenatal Care” OR “Postpartum Care” OR Childbirth OR “Pregnancy Outcomes”) AND (“Prisoners” [- Mesh] OR jail[tw] OR jailed[tw] OR prison+[tw] OR imprison+[tw] OR convict+[tw] OR felon+[tw] OR incarcerat+[tw] OR correctional[tw] OR inmate+[tw]) AND (Need+ OR Problem+ OR Requirement OR Expectation OR Perception) (Table 1).

**Table 1:** Strategy for systematic search of the published literature in Scopus database

#1	Search “Pregnancy” OR “Pregnant Women” OR “Prenatal Care” OR “Antenatal Care “OR “Postpartum Care” OR Childbirth OR “Pregnancy Outcomes”	
#2	“Prisoners” [- MeSH] OR jail[tw] OR jailed[tw] OR prison+[tw] OR imprison+[tw] OR convict+[tw] OR felon+[tw] OR incarcerat+[tw] OR correctional[tw] OR inmate+[tw]	
#3	Need+ OR Problem+ OR Requirement OR Expectation OR Perception	
#4	#1 AND #2 AND #3	
#5	Identification	Records identified through Scopus searching = 4680
#6	Screening	Records removed due to duplication = 12
		Records screened = 4668
		Records excluded based on title & abstract screening = 4578
		Non relevant = 42
#7	Eligibility	Full text articles assessed for eligibility = 48
		Full text articles excluded = 40
#8	Included	Studies included in qualitative synthesis = 8

All quantitative studies including cross-sectional, retrospective and prospective cohort, model testing, as well as case series and survey, which addressed the needs and expectations of incarcerated pregnant women, were included in this review, letters to the editor, newspapers and newsletters, conference summaries, qualitative and experimental studies were the exclusion criteria. Unrelated and duplicate papers were also excluded from the review.

Two reviewers evaluated all the articles, and the data were based on a pre-designed table (Table 2). Data included the first author’s name, year of publication, place of study, study design, sample size, types of needs cited in the results, and score of qualitative synthesis of articles. Any discrepancies between the reviewers were resolved through discussion until consensus was achieved.

**Table 2:** Descriptive summary of included studies

No.	Author/year	Title	Country	Study design	Participants (imprisoned pregnant women and control women)	The need identified	Quality of study
1.	Walker, Hilder, Levy & Sullivan 2014^50^	Pregnancy, prison and perinatal outcomes in New South Wales, Australia: a retrospective cohort study using linked health data	New South Wales, Australia	Retrospective Cohort	302 incarcerated pregnant women vs 1238 incarcerated women vs 39367 non prison women	Drug abuse management	Good
2.	Dallaire 2017^36^	A nutrition-based program for pregnant incarcerated women.	Virginia, US	Prospective Cohort	116 pregnant women with health program vs 51 pregnant women without health program	Educational & nutritional needs (Related to pregnancy and nutrition)	Average
3.	Kelsey 2017^24^	An examination of care practices of pregnant women incarcerated in jail facilities in the United States	Virginia, US	Cross sectional	106 incarcerated pregnant women	Care needs	Average
4.	Shlafer 2015^42^	Pregnancy and Parenting Support for Incarcerated Women: Lessons Learned.	Minnesota, US	Cross sectional	48 incarcerated pregnant women	Support needs	(Effective services with participation of the community, university, and prison)	Average
5.	Clark	2006^15^	Promising strategies for preventing perinatal HIV transmission: model programs from three states	Florida, US	Cohort comparison group	515 incarcerated pregnant women form 4 prisons	Care needs (Reduce vertical HIV transmission)	Average
6.	Carlson 2009^39^	A Pathway to Crime-Free Futures	Nebraska, US	Cohort disadvantaged comparison group	65 incarcerated women with prison nursery vs 30 incarcerated women	Educational needs (Parenting classes)	Average
7.	Schroeder and Bell 2005^43^	Doula birth support for incarcerated pregnant women.	Washington, US	Case series	18 incarcerated pregnant women	Support needs (Governmental supports)	Average
8.	Leifer 2003^51^	The keys to care	London, UK	Case series	120 incarcerated pregnant women	Drug abuse management (Detoxification program)	Average
9.	Mertens 2001^49^	Pregnancy outcomes of inmates in a large county jail setting.	US	Survey	71 incarcerated pregnant women	Nutritional needs	Poor
10.	Carlson 2000^40^	Prison Nursery 2000: A Five-Year Review of the Prison Nursery at the Nebraska Correctional Center for Women.24	Nebraska, US	Cohort disadvantaged comparison group	37 incarcerated women with prison nursery vs 30 incarcerated women	Educational needs (Parenting classes)	Average
11.	Siefert 2001^25^	Improving pregnancy outcome during imprisonment: a model residential care program	Washington,US	Model testing	44 incarcerated pregnant women	Care & supportive needs	Average
12.	Elton 1985^31^	Outcome of pregnancy among prisoners	Manchester, UK	Cohort	298 incarcerated pregnant women vs 298 non prison women	Care needs (Necessary care in the early stages of pregnancy)	Poor
13.	Howard 2009^32^	Timing of incarceration during pregnancy and birth outcomes: exploring racial differences.	Texas, US	Cross sectional	360 incarcerated pregnant women	Care needs (Increase the frequency of prenatal care)	Average
14.	Bell 2004^26^	Perinatal health service use by women released from jail	Washington, US	Retrospective Cohort	468 incarcerated pregnant women vs 144 non prison women	Care needs (Adequate prenatal care)	Average
15.	Tapia and Vaughn^41^	Timing of conception for pregnant women returning to jail.	Rhode Island, US	Cross sectional	269 incarcerated women	Educational needs (Postpartum classes)		Good
16.	Kyei-Aboagye 2000^33^	Birth outcome in incarcerated, high-risk pregnant women	Massachusetts, US	Retrospective Cohort	31 incarcerated pregnant women vs 71 prison women	Care needs & Drug abuse management (Midwifery care and Methadone therapy)	Average
17.	Barkauskas, Low and Pimlott 2016^35^	Health outcomes of incarcerated pregnant women and their infants in a community-based program	Midwestern Metropolitan, US		Comparative cross sectional	52 incarcerated pregnant women with Residential Program vs 73 incarcerated women	Care needs & Drug abuse management (The residential program)	Good
18.	Cordero, Hines, Shibley and Landon 1992^27^	Perinatal outcome for women in prison.	Ohio, US	Cross sectional	223 incarcerated pregnant women	Care needs (Available cares)	Poor
19.	Ferszt 2008^38^	Development of an educational/support group for pregnant	Rhode Island, Kingston, US	Cross sectional	22 incarcerated pregnant women	Educational & Consultative needs	Average
20.	Rowles 2007^39^	Birth Companions External Evaluation report	London, UK	Case series	9 incarcerated pregnant women & 5 post-partum prison women	Educational needs (Childbirth education, breastfeeding, parenting classes)	Average
21.	Eliason and Arndt 2004^53^	Pregnant inmates: a growing concern.	Iowa, US	Cross sectional	53 incarcerated pregnant women vs 1160 incarcerated women	Drug abuse management	Average
22.	Inoue 2003^44^	Models of excellence 1999–2002: Innovative Programs and Services in America’s Public Hospitals and Health Systems.	Chicago, US	Case series	50incarcerated women	Support needs	Average
23.	Martin 1997^45^	The effect of incarceration during pregnancy on birth outcomes.	North Carolina, US	Cohort	168 incarcerated pregnant women vs 3910 non prison pregnant women	Support needs (Food, shelter, clothing and cleaning)	Average
24.	Caddle and Crisp 1997^46^	Imprisoned women and mothers: Home office research study 162	UK	Case series	1766 incarcerated pregnant women or prison mother	Support needs (Not being separated from their infants)	Average
25.	Safyer 1995^29^	Pregnancy behind bars	New York, US	Cohort without comparison	group	114 incarcerated pregnant women	Care needs (Community-based prenatal care)	Poor
26.	Fogel 1993^47^	Pregnant inmates	Southern state, US	Case series	89 incarcerated pregnant women	Drug abuse management	Poor
27.	Terk, Martens, and Williamson 1993^30^	Pregnancy outcomes of incarcerated women	Texas, US	Retrospective Cohort	76 incarcerated women vs 117 non prison pregnant women	Care needs (Mandatory prenatal services)	Average
28.	Egley, Miller Granados and Ingram-Fogel,1992^52^	Outcome of pregnancy during imprisonment.	Raleigh,North Carolina, US	Cohort	69 incarcerated women vs 69 non prison pregnant women	Drug abuse management	Average
29.	Cordero, Hines, Shibley and Landon 1991^28^	Duration of incarceration and perinatal outcome.	Nevada, US	Cross sectional	53 incarcerated pregnant women with short-term sentence vs 53 incarcerated pregnant women with long-term sentence	Care & nutritional needs	Average
30.	Stauber 1984^34^	Pregnancy, labor and the puerperium in women prisoners.	Berlin, Germany	Cohort	43 incarcerated pregnant women vs 172 non prison pregnant women	Care & supportive needs (General changes in the execution of the prison sentence)	Poor
31.	Birmingham 2006^48^	The mental health of women in prison mother and baby units.	UK	Survey	4 incarcerated pregnant women	Consultative needs (Mental health problems)	Average

The methodological quality of the papers was evaluated using the combined STROBE (Strengthening the Reporting of Observational Studies in Epidemiology) statement (Table 3), which is a 22-item checklist that assesses the essential items of cross-sectional and observational studies. ^18^ It could also be applicable for case series, as any of the key elements in STROBE can be applied in this design. ^19^ The aim of the scoring method was to obtain an overall quality score for the retrieved articles. For scoring, it was decided to have scores of 0: if the particular checklist item was not fulfilled, and a score of 1: if the particular checklist item was fulfilled. Maximum possible STROBE scores for observational studies were as follows: cohort=34, case-control=33, and cross-sectional=27. ^20^ Similar studies were used to determine the cut-off point. ^21^ Obtaining 75% of the total score and above, between 25% and 75%, and less than 25% were classified as good, average, and poor quality, respectively. ^22 , 23^

**Table 3:** Qualitative synthesis of the studies using STROBE criteria

Criteria	Article number																														
	1	2	3	4	5	6	7	8	9	10	11	12	13	14	15	16	17	18	19	20	21	22	23	24	25	26	27	28	29	30	31
1a) Indicate the study design	+	-	-	-	-	-	-	-	-	-	-	-	-	+	-	+	-	-	-	-	-	-	-	-	-	-	+	-	-	-
1b) An informative and balanced summary	+	-	+	+	+	+	+	+	-	+	+	+	+	+	+	+	+	+	-	+	+	+	+	+	+	-	+	-	+	+	+
2) Background/rationale	+	+	+	+	+	+	+	+	+	-	+	+	+	+	+	+	+	+	+	+	+	+	+	+	+	+	+	+	+	+	+
3) Objectives	+	+	+	+	+	+	+	+	-	+	+	+	+	+	+	+	+	-	-	-	+	+	-	-	-	-	-	-	-	-	-
4) Study design	+	+	+	+	+	+	+	-	-	+	+	+	+	+	+	+	+	+	+	+	+	+	+	+	-	-	+	+	+	+	+
5) Setting	+	+	+	-	-	-	-	-	-	-	-	-	-	-	+	+	+	-	+	-	-	-	-	-	-	-	-	-	-	-	-
6a) Eligibility criteria, selection of participants, follow-up	+	+	+	+	+	+	+	+	+	+	+	-	+	+	+	+	+	-	+	+	-	+	+	+	+	+	+	+	+	-	+
6b) Matching criteria and number of exposed and unexposed	-	-	-	-	-	N/Aa	N/A	N/A	N/A	N/A	N/A	-	-	-	-	-	-	-	-	N/A	-	N/A	-	N/A	N/A	N/A	-	-	-	-	N/A
7) Variables	+	+	-	-	-	-	N/A	N/A	-	-	-	-	-	-	-	-	-	-	-	N/A	-	N/A	-	N/A	-	N/A	-	-	-	-	-
8) Data sources/measurement	+	-	+	+	+	+	+	+	-	+	+	-	+	+	+	+	+	+	+	+	-	+	-	+	-	-	-	-	-	-	+
9) Bias	-	+	-	-	-	-	-	-	-	-	-	-	-	-	+	-	+	-	-	-	-	-	-	-	-	-	-	-	-	-	-
10) Study size	+	-	+	+	+	+	+	+	+	+	+	+	+	+	+	+	+	+	+	+	+	+	-	+	+	+	+	+	+	+	+
11) Quantitative variables	+	+	-	-	-	+	+	+	-	-	-	-	-	-	-	-	-	-	+	-	-	-	-	-	-	-	-	-	-	-	-
12a) All statistical methods	+	+	-	-	+	+	+	+	-	+	-	+	+	+	+	+	+	+	+	+	+	+	-	+	-	-	+	+	+	-	+
12b) Subgroups and interactions	-	-	-	-	-	-	N/A	N/A	N/A	-	-	-	-	-	-	-	-	-	-	N/A	-	N/A	-	N/A	-	N/A	-	-	-	-	N/A
12c) Missing data	-	-	-	-	-	-	-	-	-	-	-	-	-	-	-	-	-	-	-	-	-	-	-	-	-	-	-	-	-	-	-
12d) loss to follow-up, matching, analytical methods	-	-	-	-	-	-	N/A	N/A	-	-	N/A	-	-	-	-	-	-	-	-	N/A	-	N/A	-	N/A	N/A	N/A	-	-	-	-	N/A
12e) Sensitivity analyses	-	-	-	-	-	-	-	-	-	-	-	-	-	-	-	-	-	-	-	-	-	-	-	-	-	-	-	-	-	-	-
13a) Numbers of individuals	+	+	+	+	+	+	+	+	+	+	+	+	+	+	+	+	+	-	+	+	+	-	-	+	+	-	+	+	+	+	+
13b) Reasons for non-participation	-	-	-	-	-	-	N/A	N/A	-	-	N/A	-	-	-	-	-	-	-	-	N/A	-	N/A	-	N/A	-	N/A	-	-	-	-	N/A
13c) flow diagram	+	-	-	-	-	-	N/A	N/A	N/A	-	N/A	-	-	-	+	-	-	-	-	N/A	-	N/A	-	N/A	-	N/A	-	-	-	-	N/A
14a) Characteristics of study participants	+	+	+	+	+	+	+	+	-	+	+	-	+	+	+	-	+	-	-	+	-	+	-	+	-	-	+	+	+	-	+
14b) Participants with missing data	-	-	-	-	-	-	N/A	N/A	N/A	-	N/A	-	-	-	-	-	-	-	-	N/A	-	N/A	-	N/A	-	N/A	-	-	-	-	N/A
14c) Summaries follow-up time	-	-	-	-	-	-	N/A	N/A	N/A	-	N/A	-	-	-	-	-	-	-	-	N/A	-	N/A	-	N/A	-	N/A	-	-	-	-	-
15) Outcome data	+	+	+	+	+	+	+	+	-	+	+	+	+	+	+	-	+	+	+	-	+	-	+	+	-	-	+	+	+	+	+
16a) Unadjusted estimates	+	+	-	-	+	+	+	-	-	+	-	-	+	+	+	+	+	-	-	-	-	+	+	+	+	-	+	+	+	+	+
16b) Category boundaries	-	-	-	-	-	-	N/A	N/A	N/A	-	N/A	-	-	-	-	-	-	-	-	N/A	-	N/A	-	N/A	-	N/A	-	-	-	-	N/A
16c) Relative risk	-	-	-	-	-	-	N/A	N/A	-	-	-	-	-	-	-	-	-	-	-	N/A	-	N/A	-	N/A	-	N/A	-	-	-	-	-
17) Other analyses	+	+	-	-	-	-	-	-	-	-	-	-	+	+	+	-	+	-	-	+	-	-	-	-	-	-	-	-	-	-	-
18) Key results	+	+	+	+	+	+	+	-	-	+	+	-	+	+	+	+	+	-	+	+	+	-	+	+	-	-	+	+	+	+	+
19) Limitations	+	+	+	-	-	+	+	-	-	-	-	-	-	-	+	-	+	-	+	-	-	-	+	-	-	-	+	+	+	+	+
20) Interpretation	+	+	+	+	-	-	-	-	-	-	+	-	+	+	+	-	+	-	+	-	-	-	-	-	-	-	-	+	-	-	+
21) Generalizability	+	+	+	+	-	-	-	-	-	-	-	-	-	-	+	-	+	-	+	-	-	-	-	-	-	-	-	-	-	-	-
22) Funding	+	+	+	-	-	+	-	-	-	+	-	-	+	+	+	-	+	-	-	-	-	-	+	+	-	-	-	-	+	-	+

## RESULTS

**Figure 1:**
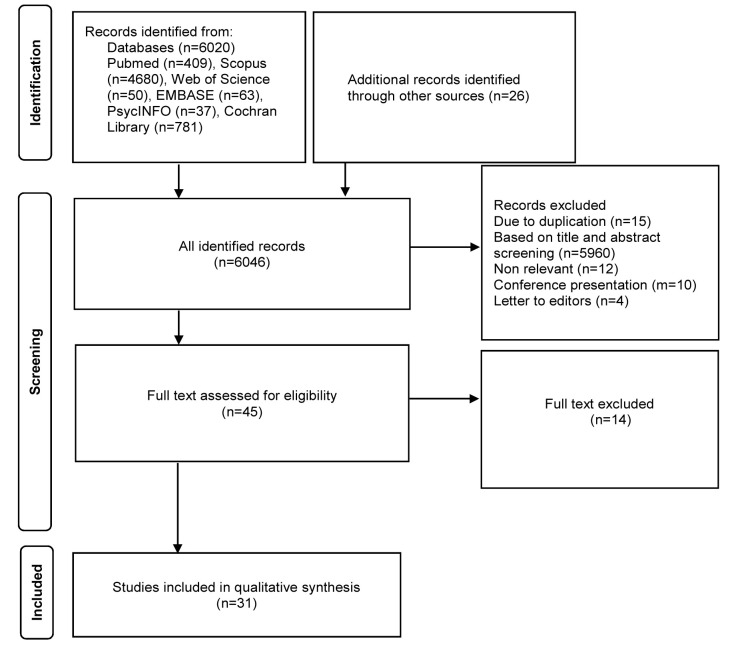
PRISMA flowchart for selection of articles

In this systematic review, initially, 6046 records were identified (6020 records identified through database searching and 26 additional records through other sources). 15 records were removed due to duplication. Thereafter, the records were screened based on the titles and abstracts, and 5960 articles were excluded. In addition, 12 records due to irrelevance, 10 articles due to being a conference abstract, and 4 articles due to being letter to editor were excluded. After reviewing the full text articles based on the inclusion and exclusion criteria for article selection, finally, 31 eligible articles were included in the qualitative assessment (Figure 1). The year of publication of the articles ranged from 1984 to 2018, and the sample size varied from four to 515 people in different studies. The total sample size was 5435 incarcerated pregnant women. 24 studies were conducted in different states of the USA, five studies in the United Kingdom, 1 study in Australia, and one study in Germany (Table 2). 

In the present study, 31 articles (9 cross-sectional studies, 13 cohort studies, 6 case series, 1 correlational model testing, and 2 surveys) were reviewed. The quality of six studies was poor, 22 average, and three good.

In these studies, the needs of pregnant women were categorized into six general categories of healthcare (13 studies), educational (6 studies), supportive (7 studies), counseling (2 studies), nutritional (3 studies), and substance abuse management (7 studies) types.

### Healthcare Needs

The need for care was concentrated on prenatal care, the care provided during labor and delivery and postpartum, the care of HIV-positive pregnant women, and prevention of vertical transmission to the fetus. The needs for care expressed in studies by Kelsey et al. (2017), ^24^ Siefert (2001), ^25^ Bell et al. (2004), ^26^ Cordero et al. (1991), ^27^ Cordero et al. (1992), ^28^ , Safyer (1995), ^29^ Terk et al. (1993) ^30^ include the need for in-prison prenatal care. Elton (1985) ^31^ points to the importance of early pregnancy care and Howard (2009) ^32^ points to the increasing number of such cares. Kyei-Aboagye (2000) ^33^ and Stauber (1984) ^34^ discussed about the need to provide care in the labor and delivery process in their article. Barkauskaset al (2016) ^35^ referred to immediate postpartum care and Clarck (2006) ^15^ referred to the care needs of HIV-positive pregnant women and prevention of vertical transmission to the fetus. 

### Educational Needs

Educational needs were discussed in relation to pregnancy classes, childbirth classes, classes for a doula, parenting classes, as well as family planning and safe sex counseling. 

The educational needs mentioned in the studies of Dallaire (2017), ^36^ Carlson (2009) ^37^ and Ferszt (2008) ^38^ were about the concerns related to pregnancy. Rowles (2007) ^39^ and Carlson (2000) ^40^ considered the educational needs during childbirth, parenting skills, and infant care as important needs. On the other hand, Tapia and Vaughn (2010) ^41^ indicated the need of these women to education on postpartum pregnancy follow-up methods.

### Supportive Needs

The review of the included studies showed that one neglected need of incarcerated pregnant women was support. The support needs were discussed in some areas including organization support, labor and delivery support, need for social workers, release from prison, and separation from their baby.

Regarding the support needs, Stauber (1984) ^34^ and Shlafer et al. (2015) ^42^ referred to the support of the prison authorities of pregnant prisoners. Schroeder and Bell (2005) ^43^ and Inoue (2003) ^44^ discussed the need for pregnant women to have double support during labor and delivery. The need for a social worker is mentioned in Siefert (2001) ^25^ in prison and Martin (1997) ^45^ after release from prison. Caddle and Crisp (1997) ^46^ pointed to the mother prisoner’s need for support during separation form her infant.

### Counseling Needs

Psychological disorders, drug abuse, the stress of pregnancy and childbirth in prison, and most importantly, separation from the child are among the issues that indicate the need for counseling services to pregnant women. Promoting the health of pregnant women in prison, including mental health, is one of the needs mentioned in the study of Fogel (1993) ^47^ and Brimingham et al. (2006). ^48^

### Need for Nutrition, Activity, and Rest

Nutritional needs, and also need to adequate activity and rest for pregnant women in prison, were also mentioned in the studies by Cordero et al. (1991), ^28^ Mertens (2001) ^49^ and Dallaire (2017). ^36^

### Need Related to Substance Abuse Management

Substance abuse management, methadone therapy, and addiction withdrawal planning are among the needs of incarcerated pregnant women in the studies of Walker et al. (2014), ^50^ Leifer (2003), ^51^ Kyei-Aboagye (2000), ^33^ Barkauskas et al. (2016), ^35^ Egley et al. (1992) ^52^ and Eliason and Arndt (2004). ^53^ According to Fogel (1993), ^47^ 90% of incarcerated pregnant women have a history of drug or psychotropic substance abuse.

## DISCUSSION

This was a systematic review to determine the needs of pregnant women in prisons. This review summarized the evidence from thirty-one studies. The results showed that the needs of incarcerated pregnant women consisted of six general categories of healthcare, educational, supportive, counseling, nutritional, and substance abuse management types.

Despite the efforts made, there are still many deficiencies in the field of pregnant women’s health in prison. It is considered necessary to recognize and update the needs of this vulnerable group of women in order to provide comprehensive and complete care programs. ^14^

One of the needs of incarcerated pregnant women was healthcare. It has been reported that less attention to incarcerated pregnant women’s needs has more costs for society. The reason is that it could be associated with adverse physical and psychological effects. ^4^ Some studies have shown the need for prenatal care models with a focus on specific healthcare needs of pregnant inmates, while there is limited information about different types of care models for this population. ^16 , 54 , 55^ The American College of Obstetricians and Gynecologists has emphasized that to meet the need for prenatal care in prisons, the care should be provided in accordance with the academic guidelines and should not be less than what is provided in the community. ^56^ However, evidence shows that health and well-being needs are not met in most prisons. ^57^

Another need of incarcerated pregnant women was need to education. It has been argued that education regarding pregnancy and parenting is one of the oldest and most detailed health promotion needs. ^53^ Some studies have shown that incarcerated pregnant women, particularly, need education as they usually experience high-risk and complex pregnancies due to their history, such as drug and alcohol abuse, high-risk sexual behaviors, and related diseases. ^3 , 58 , 59^ Consistent with our findings, the results of a systematic review showed that a safe and secure prison environment could be a good place to teach parenting skills to pregnant women. ^12^ Also, it was highlighted by another review article that through educating during pregnancy, it would be possible to change the behaviors and lifestyle of women in order to provide a positive attitude towards pregnancy and childbirth. ^4^

Supportive needs were another neglected need of incarcerated pregnant women in this review. It is obvious that pregnant women and their infants are among the population that need supportive community programs. ^60^ In line with our results, it was reported in a study that an early intervention framework using external support can have a significant supportive impact on pregnant women and their families. ^61^ Some studies suggested that the need for support is especially vital in pregnant women, particularly, financial support. In addition, the need for emotional support seems vital for pregnant women in prison due to being away from families and friends. Furthermore, social support could help incarcerated pregnant women to cope with the crisis of being jailed. ^62 - 65^ It has also been reported that many of these women have no contact with the outside world during pregnancy and childbirth and do not receive support from family or friends, so they need more supportive cares. ^66^ However, in one study in New South Wales, Australia, it was found that prisons did not provide the social and even emotional support needed. ^51^ It has been emphasized in a review article that one of the sources of support is adoption of organizational policies and laws that support pregnant women and lead to the establishment of reproductive justice for mothers and the protection of their infants and children in prison. ^13^ In another study which analyzed the Washington State’s Residential Parenting Program for Pregnant Inmates, it was stressed that prison can provide a great opportunity to reach a vulnerable group of pregnant women and, through provision of the right health and social services, promote their health in prison and their ability to participate in community-based programs even after release. ^58^

Another need of incarcerated pregnant women was their need for counseling. Researchers attribute the need for counseling to the stressful and painful life of pregnant prisoners, which can have a devastating effect on their well-being and health. In addition, they believe that incarceration hinders the pregnant women’s ability to adapt. ^65 , 66^ However, a survey of all US prisons with a 61 percent response rate found that there was no significant counseling program to address the mental health problems of pregnant inmates However, some prisons offer counseling programs for all female prisoners and particularly have special programs tailored to the needs of pregnant women. ^67^ It has been found that there is a lower rate of cases of psychosis and neurological disorders in pregnant prisoners receiving psychological counseling, so prison can become a place for treatment. As a result, screening, identifying and treating mental disorders can benefit many vulnerable mothers in this population. ^49^ Counseling and correcting high-risk behaviors can play a unique role in improving pregnancy outcomes, so that, according to a study (1993), women who spend more weeks in prison, while receiving more counseling services, had better pregnancy outcomes. ^30^

One of the most important and neglected needs of incarcerated pregnant women was poor nutrition during pregnancy. One study reported that women who received high quality foods during imprisonment experienced less maternal and fetal complications than others. ^68^ In another interventional study which investigated the effect of a nutrition-based program on incarcerated pregnant women, it was concluded that because of dietary changes during pregnancy, the criminal justice system could use this opportunity to develop a nutritional support program for vulnerable and disadvantaged groups. ^36^ Therefore, researchers have provided recommendations for improving the nutritional care of incarcerated pregnant women in accordance with the guidelines provided by the Academy of Nutrition and Dietetics. ^36 , 69 , 70^ In this way, the limited opportunity of imprisonment can be used to provide nutritional education to this population in order to have benefits beyond imprisonment. In another study which examined low birth weight (LBW) and fetal death rates for women incarcerated during pregnancy, it was concluded that the role of nutrition in promoting health and improving the consequences of pregnancy in female prisoners is very important basic principle. ^50^

A study which compared demographic features and substance use in pregnant and non-pregnant female inmates reported that it was important to identify the potential needs of these women in order to improve their conditions in prison and subsequently improve the outcome of pregnancy and childbirth. ^53^ In a narrative review, which focused on the international guidelines for incarcerated pregnant women, it is suggested that corrective facilities that care for these women should be based on standards of care and evidence-based methods to treat women with methadone or buprenorphine. It is also recommended that these centers should provide the necessary facilities for care at the time of consumption in order to meet the medical needs of the pregnant women and their fetus and prevent serious problems. ^14^ Appropriate counseling and opioid antagonistic drug treatments are among the suggested solutions to incarcerated pregnant women who are addicts. ^53^

One of the strengths of this study was using an extensive search strategy to find the relevant studies. However, like all studies it had some limitations. Most of the studies were conducted in the United States, which limits the generalizability of the findings. In addition, no information was obtained from middle- and low-income countries. In addition, the results of some studies did not follow the coherence and logical order, and this forced us to classify the results. In this review, only peer-reviewed articles were included, which could increase the risk of publication bias. 

## CONCLUSION

This study is the first systematic review of the needs of incarcerated pregnant women. Recognition of these needs and trying to meet them could improve the health of pregnancy in prison and improve its long-term and short-term results. In general, the needs of pregnant women in prison include the needs for adequate and quality prenatal care; education on pregnancy, childbirth and parenting, psychological and supportive counseling, nutrition, and substance abuse management. By recognizing these needs, accurate and appropriate programs can be designed and implemented for this vulnerable group. This systematic review showed evidence of a set of needs of incarcerated pregnant women that can be further investigated in future studies. In addition, this study could give insight to future researchers to explore other neglected needs of incarcerated pregnant women.

## ACKNOWLEDGEMENT

The present study is part of the results of the Reproductive Health doctorate thesis of the first author (SA), approved by Mashhad University of Medical Sciences with the code of 980109 and ethics committee code of IR.MUMS.REC.1398.099. The financial support of Mashhad University of Medical Sciences is warmly appreciated.

**Conflict of Interest:** None is declared. 
